# Closing the Equity Gap of Access to Emergency Departments of Private Hospitals in Thailand

**DOI:** 10.1155/2018/6470319

**Published:** 2018-09-26

**Authors:** Paibul Suriyawongpaisal, Pongsakorn Atiksawedparit, Samrit Srithamrongsawad, Thanita Thongtan

**Affiliations:** ^1^Department of Community Medicine, Faculty of Medicine, Ramathibodi Hospital, Mahidol University, Rama 6, Ratchathewi, Bangkok, Thailand; ^2^Department of Emergency Medicine, Faculty of Medicine, Ramathibodi Hospital, Mahidol University, Rama 6, Ratchathewi, Bangkok, Thailand; ^3^Department of Physiology, Faculty of Science, Mahidol University, 272 Rama 6, Ratchathewi, Bangkok 10400, Thailand

## Abstract

**Background:**

Previous policy implementation in 2012 to incentivize private hospitals in Thailand, a country with universal health coverage, to provide free-of-charge emergency care using DRG-based payment resulted in an equity gap of access and copayment. To bridge the gap, strategic policies involving financial and legal interventions were implemented in 2017. This study aims to assess whether this new approach would be able to fill the gap.

**Methods:**

We analyzed an administrative dataset of over 20,206 patients visiting private hospital EDs from April 2017 to October 2017 requested for the preauthorization of access to emergency care in the first 72 hours free of charge. The association between types of insurance and the approval status was explored using logistic regression equation adjusting for age, modes of access, systolic blood pressure, respiratory rate, and Glasgow coma scores.

**Results and Discussion:**

The strategic policies implementation resulted in reversing ED payer mix from the most privileged scheme, having the major share of ED visit, to the least privileged scheme. The data showed an increasing trend of ED visits to private hospitals indicates the acceptance of the financial incentive. Obvious differences in degrees of urgency between authorized and unauthorized patients suggested the role of preauthorization as a barrier to the noncritical patient visiting the ED. Furthermore, our study depicted the gender disparity between authorized and unauthorized patients which might indicate a delay in care seeking among critical female patients. Lessons learned for policymakers in low-and-middle income countries attempting to close the equity gap of access to private hospital EDs are discussed.

## 1. Introduction

Since the passage of the Patient Protection and Affordable Care Act (ACA) in the US in 2010, evidence indicates changes in emergency department (ED) visits and ED payer mix. Total ED use per 1,000 population increased by 2.5 visits more in Medicaid expansion states than in nonexpansion states after 2014 [1]. In contrast, using diagnosis-related group (DRG)-based payment in 2012 to incentivize private hospitals to provide ED services without patient copayment in Thailand resulted in disproportionately higher access by Civil Servant Medical Benefit Scheme (CSMBS) patients than the other two major public health insurance patients [2]. Although CSMBS beneficiaries constitute just 8.6% of all public health insurance beneficiaries, 59.8% of all the patient's access to the ED services was CSMBS patients. To address this inequitable ED access, three strategic policies were established in 2017 directed at private hospitals under the Universal Coverage for Emergency Patients (UCEP). The strategic policies are (a) a legal requirement precluding private hospitals from charging the patients during the first 72 hours of ED services, (b) a preauthorization of access for patients in critical conditions, and (c) fee-schedule payment to private hospitals out of negotiation among policymakers, regulator, private hospitals, and the public health insurance agencies.

Eligibility of access to ED services in the first 72 hours without copayment is determined by preauthorization processes set up by the National Institute of Emergency Medicine (NIEMS). Chief complaints were categorized into 25 groups (20 nontraumatic and 5 traumatic), and physical examination includes blood pressure (BP), respiratory rate (RR), Glasgow coma score (GCS), and oxygen saturation. If chief complaint/initial evaluation meet at least one criteria of Emergency Severity Index (ESI) levels 1 and 2, or the patient has any of the following signs: unresponsiveness, cardiac arrest, hypotension, respiratory failure, oxygen desaturation, stroke, or myocardial infarction, that patient will be automatically authorized. In case of an inconclusive result, an assigned emergency physician (EP) will be consulted to give the final say. The EP is 24 hours available and appointed by the National Institute of Emergency Medicine (NIEMS) which is responsible for the development and execution of the preauthorization program. This study aims to assess whether implementing the policies would result in improved equity in ED access.

## 2. Methods

Administrative datasets among 20,206 patients visiting private hospital EDs from April 2017 to October 2017, after requesting for the preauthorization, were analyzed. Patient information included demographics (region of residence, types of insurances, age, and gender) and clinical characteristics (chief complaints and triage level). Patient discharge status of 3,355 authorized patients from April 2017 to June 2017 was initially reviewed.

Categorical data were described as number and percentage, whereas continuous data were represented as mean with standard deviation (SD) or median with interquartile range when appropriate. Association between types of insurance and the approval status was explored using logistic regression equation adjusting for age, modes of access, systolic blood (SBP), RR, and GCS. Statistical analysis was performed using Stata version 14.2 (Stata Corp, College Station, Texas, USA). P values less than 0.05 were considered statistically significant.

This study was approved by Ethical Review Board of the Faculty of Medicine, Ramathibodi Hospital (ID 10-60-30: An assessment of a Policy on Universal Coverage of Emergency Patients (UCEP), November 2017).

## 3. Results

Demographic characteristics of patients are summarized in Table 1; over half of the patients were Bangkok residents where private hospitals are most abundant in the country. Universal Coverage Scheme (UCS) patients, the most disadvantaged and largest group of all public health insurance patients, had the biggest share of ED access (approximately 60%). With a balanced gender composition, there was no evidence of gender inequality of access to private hospital EDs. However, preauthorization resulted in the higher proportion of male patients eligible for access to the first 72 hours of emergency care (Table 2). This gender disparity remains after adjusting for confounding factors as shown in Table 3. Among the top ten ED chief complaints (85% of all the patients) (Table 4), the majority were medical emergency conditions which corresponded to the mean age of 55 years in our studied population.

**Figure 1:**
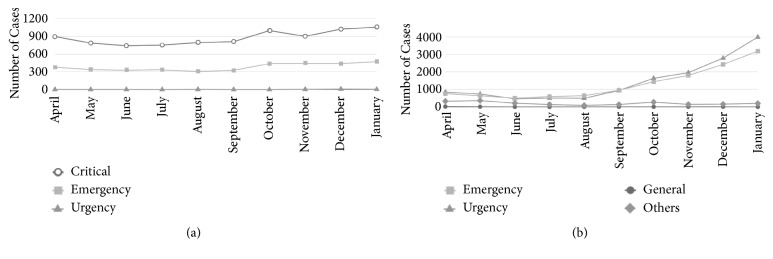
The trend of ED visits categorized by urgency levels in authorized patients (a) and unauthorized patients (b) from April 2017 to October 2017.

Obvious differences in degrees of urgency between authorized patients and unauthorized patients are depicted in Table 5. This data indicates the effectiveness of preauthorization mechanism as a barrier for those that were not deemed to be critical. Furthermore, Figure 1 shows a more remarkable increasing trend of visits to EDs in patients without approval (panel (b)).

Multivariate analysis confirmed the findings in Tables 1 and 2 that UCS patients were more likely to be authorized compared with their counterparts (Table 6). Given that almost 40% of records on discharge status from ED among patients with approval were missing, this raised a concern about data quality (Table 7). Tabulation of the available data for the discharge status revealed that the majority of the patients (~56%) were either hospitalized or transferred to more advanced hospitals. This finding corroborated with those in Table 5; the authorized patients were more critically ill.

## 4. Discussion

Analysis of preauthorization dataset clearly showed that the strategic policies implementation resulted in reversing payer mix of the patients from the most privileged scheme (CSMBS) having the major share of ED visit, according to the previous study [2], to the least (UCS) in the present study (Tables 1 and 5). Our findings support the effects of ACA's Medicaid expansion on changes in payer mix (reduction in the uninsured share of ED visits in Medicaid expansion states compared with nonexpansion states [1]).

Increasing trend of ED visits to private hospitals as opposed to that of the previous study (declining trend of reported ED visits [2]) indicates the private hospitals' acceptance of the financial incentive. Exploring working mechanisms of the incentive may offer a more interesting lesson for policymakers. Three main reasons could explain this finding. First, a higher compensation rate for private hospitals (about 50% of hospital charge) was adopted under the current policy as compared to the previous one (34% of hospital charge [3]). Second, the detailed formulation of the payment scheme was undertaken in consultation with private hospitals which was not previously the case [2]. Finally, the establishment of the legal obligation at present may act in synergy with the other two strategies. The effectiveness of the law might be attributed to close engagement with private hospitals by the Health Minister at the commencement of the policy implementation and an official complaint system for user led to a case-by-case investigation of private hospital compliance conducted by the Ministry of Public Health and NIEMS [4].

The gender disparity of access to the first 72-hour services in our study is in keeping with earlier reports from high-income countries regarding lower chances of access to acute care for stroke or coronary syndromes by female patients compared to male patients [5, 6]. This gender disparity might indicate delay in care seeking among female patients as shown by a previous study which depicted that risk of delay in hospital arrival was 3 times greater in women than in men [7]. According to this same study, perceptual, social, and behavioral factors contribute to delay in seeking medical care in acute ischemic stroke beyond demographic and clinical variables, and, when combined, further increase the risk of delay.

Despite this seeming success in partially closing the equity gap of access, a substantial number of patients (44% with emergency medical conditions, in Table 5) were denied approval as a result of preauthorization, which raises a concern about patient safety. Using the need of hospitalization as a measure of safety, a systematic review did not find sufficient evidence for the validity of 5 triage scales including ESI [8]. Furthermore, substantial missing data (almost 40%, Table 6) on discharge status from the EDs raises a concern about the deficit in monitoring mechanism to ensure access to definitive care.

Further research is needed to follow up on outcomes of definitive care among patients with approval and on whether those without approval would access timely appropriate care. More studies are also worthwhile to explore factors contributing to the gender disparity.

## 5. Limitations

The substantial missing of data for discharge status from EDs may compromise our ability to reliably assess the extent of patients' access to definitive care. However, the fact that less than 1% of the patients were discharged home may lessen a concern of underestimation of access to definitive care.

## Figures and Tables

**Table 1:** Summary of patient demographics requested for the preauthorization of access to ED service in the first 72 hours without copayment (n = 20,206).

**Characteristics**	**No.**	**(**%**)**
**Region**		
Bangkok	10,820	(53.56)
Middle	2,196	(10.88)
East	1,866	(9.24)
West	1,743	(8.63)
North East	1,734	(8.58)
North	1,090	(5.40)
South	751	(3.72)
Total	20,200	(100)
**Health insurance status**		
UCS	11,121	(56.21)
CSMBS	5,444	(27.52)
SSS	2,847	(14.39)
Others	373	(1.89)
Total	19,785	(100)
**Age (years), mean (SD)**	54.55	(24.20)
**Gender**		
Male	10,311	(51.49)
Female	9,713	(48.51)
Total	20,024	(100)

**Table 2:** Selected characteristics of authorized and unauthorized patients.

**Characteristics**	**Authorized patients**				**Unauthorized patients**		**P-value**
	**ESI 1**		**ESI 2**				
	**No.**	**(**%**)**	**No.**	**(**%**)**	**No.**	**(**%**)**	
**Health insurance status**							< 0.001
CSMBS	1,193	(20.43)	430	(18.36)	3,821	(32.93)	
SSS	671	(11.49)	352	(15.03)	1,824	(15.72)	
UCS	3,923	(67.19)	1,524	(65.07)	5,674	(48.90)	
Others	52	(0.89)	36	(1.54)	285	(2.46)	
Total	5,839	(100)	2,342	(100)	11,604	(100)	
**Gender**							< 0.001
Male	3,293	(55.04)	1,511	(64.05)	5,507	(47.14)	
Female	2,690	(44.96)	848	(35.95)	6,175	(52.86)	
Total	5,983	(100)	2,359	(100)	11,682	(100)	

**Table 3:** Univariate and multivariate analysis of the association between gender and approval status.

**Gender**	**Univariate analysis**			**Multivariate analysis** ^**∗**^		
	**OR**	**(95**%** CI)**	**P-value**	**OR**	**(95**%** CI)**	**P-value**
Male	1.52	(1.43, 1.61)		1.67	(1.55, 1.80)	<0.01
Female	1			1		

^*∗*^Adjusted by age, gender, modes of access, systolic blood pressure, respiratory rate, and Glasgow coma scores.

**Table 4:** Top ten ED chief complaints.

**Chief complaint**	**No.**	**(**%**)**
Fatigue	3,376	(16.71)
Dyspnea	3,005	(14.87)
Chest pain/discomfort	2,097	(10.38)
Paralysis	1,803	(8.92)
Road traffic injury	1,570	(7.77)
Abdominal/back/pelvic pain	1,469	(7.27)
Unresponsiveness	1,116	(5.52)
Falling	1,008	(4.99)
Cardiac arrest	842	(4.17)
Seizure	725	(3.59)
Others	3,195	(15.81)
Total	20,206	(100)

**Table 5:** Level of urgency in the authorized and unauthorized patients.

**Triage level**	**Authorized patients**				**Unauthorized patients**	
	**ESI level 1**		**ESI level 2**			
	**No.**	**(**%**)**	**No.**	**(**%**)**	**No.**	**(**%**)**
Critical	5,907	(97.59)	0	(0)	0	(0)
Emergency	142	(2.35)	2,376	(99.96)	5,225	(44.37)
Urgency	4	(0.07)	1	(0.04)	5,041	(42.81)
General	0	(0)	0	(0)	1,485	(12.61)
Others	0	(0)	0	(0)	25	(0.21)
Total	6,053	(100)	2,377	(100)	11,776	(100)

**Table 6:** Univariate and multivariate analysis of the association between health insurance status and approval status.

**Health insurance status**	**Univariate analysis**			**Multivariate analysis** ^*∗* ^		
	**OR**	**(95**%** CI)**	**P-value**	**OR**	**(95**%** CI)**	**P-value**
UCS	2.26	(2.1, 2.42)	< 0.01	2.33	(2.12, 2.55)	< 0.01
SSS	1.32	(1.19, 1.45)	< 0.01	1.77	(1.56, 2.02)	< 0.01
CSMBS	1			1		

^*∗*  ^Adjusted by age, gender, modes of access, systolic blood pressure, respiratory rate, and Glasgow coma scores.

**Table 7:** ED discharge status of authorized patients.

**Discharge status**	**No.**	**(**%**)**
ICU admission	1,000	(27.62)
Ward admission	648	(17.90)
transferred	367	(10.14)
Death at ED	195	(5.39)
Discharge home	20	(0.55)
Deny treatment	15	(0.41)
Missing data	1,375	(37.98)
Total	3,620	(100)

## Data Availability

Datasets used in this report may be requested from the authors.
